# Non‐Thermal Plasma in Contact with Water: The Origin of Species

**DOI:** 10.1002/chem.201503771

**Published:** 2016-02-02

**Authors:** Yury Gorbanev, Deborah O'Connell, Victor Chechik

**Affiliations:** ^1^ Department of ChemistryUniversity of YorkHeslington, YorkYO10 5DDUK; ^2^ York Plasma InstituteDepartment of PhysicsUniversity of YorkHeslington, YorkYO10 5DQUK

**Keywords:** plasma chemistry, EPR spectroscopy, isotopic labelling, radicals, reactive oxygen species

## Abstract

Non‐thermal atmospheric pressure plasma has attracted considerable attention in recent years due to its potential for biomedical applications. Determining the mechanism of the formation of reactive species in liquid treated with plasma is thus of paramount importance for both fundamental and applied research. In this work, the origin of reactive species in plasma‐treated aqueous solutions was investigated by using spin‐trapping, hydrogen and oxygen isotopic labelling and electron paramagnetic resonance (EPR) spectroscopy. The species originating from molecules in the liquid phase and those introduced with the feed gas were differentiated by EPR and ^1^H NMR analysis of liquid samples. The effects of water vapour and oxygen admixtures in the feed gas were investigated. All the reactive species detected in the liquid samples were shown to be formed largely in the plasma gas phase. It is suggested that hydrogen peroxide (determined by UV/Vis analysis) is formed primarily in the plasma tube, whereas the radical species ⋅OOH, ⋅OH and ⋅H are proposed to originate from the region between the plasma nozzle and the liquid sample.

## Introduction

Non‐thermal plasmas have attracted increased attention in recent years due to their potential for biomedical applications.^1^, ^2^, ^3^, ^4^ The interaction of these plasmas with ambient atmosphere results in the formation of a variety of reactive species that exhibit high biological activity (e.g., anti‐microbial, anti‐cancer, wound healing).^5^, ^6^, ^7^, ^8^, ^9^ A range of spectroscopic techniques have been used to monitor different reactive species in these plasmas, for example, IR optical emission spectroscopy for ^1^O_2_,^10^ diode laser absorption spectroscopy for metastable states of helium,^11^ vacuum ultraviolet (VUV) absorption spectroscopy and laser‐induced fluorescence for radical and atomic species,^12^, ^13^ FTIR spectroscopy for hydrogen peroxide^14^ and mass‐spectrometry for ionic species.^15^

Aqueous media is a fundamental part of the biological milieu. The two main types of plasma applications in research and biomedical trials are the pre‐treatment of aqueous media, which is subsequently applied to tissue or bacteria, and the direct exposure of biological samples to a plasma jet.^16^, ^17^ Whereas the first method relies on the formation of relatively long‐lived reactive species, such as hydrogen peroxide and ozone, as well as secondary radicals generated in the liquid phase,^18^ the efficacy of the latter is dependent on short‐lived species such as ^1^O_2_ and radicals including the hydroxyl radical, ⋅OH, the superoxide radical, O_2_⋅^−^, and atomic radicals directly formed by the plasma. Investigating the factors that govern the formation of reactive species in plasma‐treated liquids is therefore very important for biomedical applications.

Electron paramagnetic resonance (EPR) spectroscopy is the most direct method of radical detection in a liquid. Short‐lived radical species are usually detected in liquid solutions by using spin traps.^19^ Tani et al. and Takamatsu et al. described the detection of radical species in plasma‐treated liquids by using various spin traps.^20^, ^21^ The concentrations of the ⋅OH and ⋅OOH radical adducts of 5‐*tert*‐butoxycarbonyl‐5‐methyl‐1‐pyrroline *N*‐oxide (BMPO) and 5,5‐dimethyl‐1‐pyrroline *N*‐oxide (DMPO) spin traps were measured in liquid samples by Reuter and co‐workers.^22^ Uchiyama et al. performed EPR and flow cytometric studies of free radicals induced in liquid by non‐ thermal plasma.^23^ In many reports, it has been proposed that ⋅OH and other radicals are at least partially formed from the dissociation of the liquid phase molecules.^21^

The concentrations of stable molecules in plasma‐treated liquids have also been measured. Reuter and co‐workers assessed the concentration of H_2_O_2_ in the liquid phase as a function of feed gas humidity by microscope analysis of colour test stripes.^14^ The authors found a direct correlation between the concentration of H_2_O_2_ in the liquid media and in the gas phase (the latter was measured by FTIR spectroscopy). Lukes et al. determined H_2_O_2_ concentration in the liquid phase colourimetrically by using titanium sulfate.^24^

Recently, Xu et al. measured concentrations of H_2_O_2_, O_2_⋅^−^, ⋅OH and ⋅H in argon plasma‐treated liquid samples containing cell cultures (colourimetrically and by using EPR spectroscopy).^9^ The authors proposed the in situ formation of the hydroxyl radical from hydrogen peroxide and the superoxide radical anion catalysed by iron‐containing proteins and correlated the hydroxyl radical formation with induced cell death. Reports of the identification of reactive species in plasma‐treated liquids, however, remain relatively scarce, and our understanding of where the reactive species originate from and how their concentrations depend on experimental parameters is limited.^25^

Several computational approaches have been used to model the reactive species in plasmas. For example, a global model for discharges in helium with admixtures of H_2_O was described by Bruggeman and co‐workers.^26^ Murakami et al. developed models of the chemical kinetics^27^ and afterglow (effluent) chemistry for helium plasma with oxygen admixtures and water vapour impurity.^28^ Kushner and co‐workers presented a model in which plasma effluent was in contact with liquid water.^29^ The authors described the formation and distribution of reactive species in a plasma effluent in contact with a liquid sample. More recently, Lindsay et al. predicted the distribution of reactive species in liquid treated by plasma by using a neutral mass transport model for convective gaseous plasma/liquid water systems.^30^ In general, plasmas in contact with liquids are extremely complex systems, making modelling very challenging. Kinetic models sometimes include hundreds of rate coefficients obtained from the literature. The accuracy of the modelling thus relies on the accuracy of the original data. At the plasma/liquid interface, other factors such as diffusion coefficients, sample evaporation and convection must be considered. Therefore, further experimental work is needed to improve our understanding of plasma/liquid systems as well as benchmark models and simulations.

In this report we present the results of an experimental study of the origin of the reactive species induced in a liquid sample by non‐thermal plasma treatment. The effect of H_2_O and O_2_ admixtures in plasma feed gas on the generation of ⋅OH, O_2_⋅^−^, O_3_ and H_2_O_2_ in plasma‐treated liquid samples was investigated. These data, and the use of isotopically labelled water (H_2_O/H_2_^17^O, H_2_O/D_2_O), has allowed us to distinguish between the species generated in the liquid phase, those that diffused into the liquid from the plasma gas phase and those formed either in the plasma core or close to the gas/liquid interface.

## Results and Discussion

### Experimental setup

In biomedical applications of cold plasma, tissue or bacteria are exposed to plasma in the ambient atmosphere. The interaction between plasma and air leads to the formation of species that introduce additional reaction pathways and post‐discharge reactions.^6^, ^24^, ^31^, ^32^ To elucidate the origin of the reactive species in solution, a simplified system is required to exclude uncontrollable local concentrations of oxygen, nitrogen, water vapour and other components of the ambient atmosphere.

**Figure 1:**
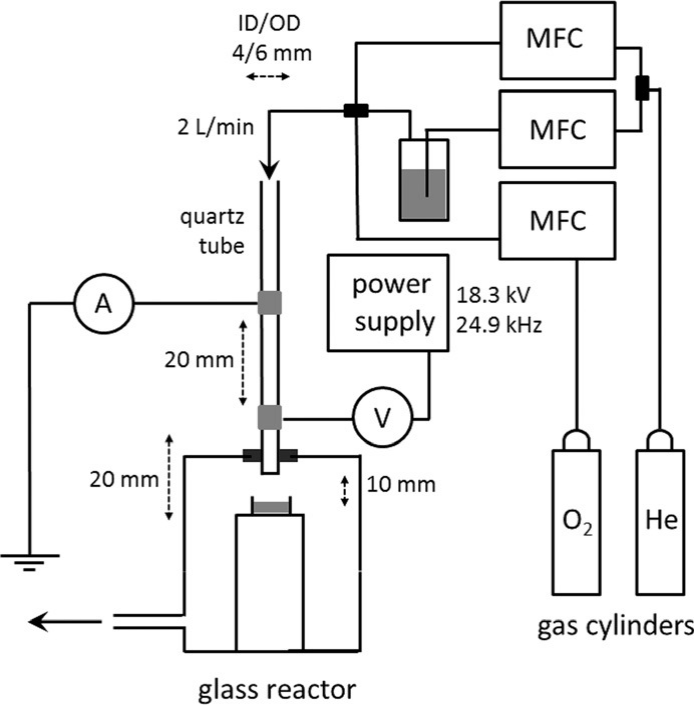
Setup used in the plasma exposure experiments. Feed gas flow was controlled by the mass flow controllers (MFCs). In all experiments the distance between the nozzle and the sample was 10 mm unless stated otherwise.

In the investigation presented herein, an in‐house‐designed reactor was used in which the plasma jet was in direct contact with the liquid (aqueous) sample (Figure 1). The atmosphere inside the reactor was composed exclusively of the plasma feed gas and additional vapour originating from the evaporation of the liquid sample. Sample evaporation was observed even at 100 % humidity of the feed gas, which suggests that the temperature of the feed gas (20 °C at the inlet) increased in the plasma reactor. Indeed, the temperature of the liquid sample measured immediately after plasma exposure was 24 and 26.8 °C for the dry and water‐saturated helium, respectively.

The plasma used was a parallel field kHz frequency jet. The plasma was ignited in a quartz tube with two copper electrodes positioned around the tube and operated with a sinusoidal voltage of about 18 kV. Helium was used as carrier gas with molecular admixtures. A detailed experimental description can be found in the Supporting Information.

Reactive species in the plasma‐treated liquids could form not only through reactions with the gas‐phase constituents of the plasma, but also through photolysis by plasma‐emitted UV and VUV photons.^33^ To test whether UV/VUV irradiation of our setup affects the formation of reactive species in the liquid sample, control experiments were carried out in which the sample was covered with a MgF_2_ window (Crystran Ltd., >40 % transmittance at 121 nm) and then exposed to plasma. In these experiments, the surface of the sample was in direct contact with the window to prevent UV quenching in the gas between the window and the sample (helium‐operated plasma is transparent to UV). The results obtained (see Figure S4 in the Supporting Information) revealed that neither radicals nor hydrogen peroxide were formed in the liquid due to photolysis reactions.

For reactive species formed in the plasma gas phase (e.g., in the plasma core), the efficiency of plasma treatment critically depends on the diffusion of these species into the liquid. To assess the diffusion properties of our setup, we investigated the delivery of molecules from the gas phase into the liquid sample by using isotopically labelled water. In these experiments, the delivery of H_2_O vapour into a liquid sample of D_2_O (and vice versa) was studied by ^1^H NMR spectroscopy with sodium tosylate as an internal standard. The use of isotopes made it possible to distinguish the water molecules originally present in the sample (D_2_O) from those delivered by the plasma (H_2_O). The results are presented in Table 1.

**Table 1:** Amount of H_2_O delivered by helium flow to a liquid D_2_O sample over a 60 s exposure time

Entry	Relative humidity (H_2_O) of the feed gas [%]	H_2_O delivered to the liquid sample^[a]^ [mol %]
**Plasma OFF**		
1	0^[b]^	<0.05
2	10	0.7
3	100	5.3
**Plasma ON**		
4	0^[b]^	0.1
5	10	1.7
6	100	13.5
7^[c]^	10	1.8
8^[c]^	100	13.1
9^[d]^	100^[d]^	13.8^[e]^

[a] The data are corrected for the initial concentration of H_2_O in the D_2_O sample and the amount of H_2_O introduced through the handling of the sample (ca. 0.2 mol % altogether). [b] Feed gas contained trace amounts of water vapour. [c] Data obtained at a distance from the nozzle to the sample of 4 mm. [d] Data obtained by using a H_2_O liquid sample and D_2_O vapour in the feed gas. [e] D_2_O delivered to the liquid sample of H_2_O.

The amount of H_2_O introduced into the sample during the setup of the experiment was subtracted from the recorded data. This amount was measured experimentally when a 100 μL sample of D_2_O was placed in a well on top of the glass stand inside the reactor pre‐flushed with helium. The sample was kept for 80 s without exposure to plasma or gas flow, after which it was analysed by ^1^H NMR spectroscopy.

With the dry helium feed gas, a small amount of H_2_O was delivered into the liquid sample (Table 1, entries 1 and 4). A clear increase in the amount of H_2_O in D_2_O was observed after a prolonged plasma exposure time (see Figure S5 in the Supporting Information). This is likely due to the physisorbed water from the gas tubing^34^ or H_2_ and H_2_O impurities present in the feed gas.

The introduction of water vapour into the feed gas clearly increased the amount of H_2_O delivered into the liquid D_2_O sample. This increase was observed under both plasma‐off (Table 1, entries 1–3) and plasma‐on conditions (entries 4–6). The system with the plasma ignited exhibited an enhanced delivery of water vapour into the liquid sample. This can be ascribed to several factors, including temperature increase and turbulent flow of the plasma jet.^35^, ^36^

To confirm the validity of these results, a control experiment was carried out with a H_2_O liquid sample exposed to plasma operated with D_2_O‐saturated feed gas. The amount of D_2_O delivered into the liquid H_2_O sample was very similar to the amount of H_2_O delivered into the liquid D_2_O sample (Table 1, entries 6 and 9).

We were interested to see whether the delivery of molecules from the gas into the liquid is influenced by the distance between the nozzle and the sample. This was tested by comparing the delivery of H_2_O into the sample located at two different distances under the nozzle: 10 and 4 mm. The distance of 4 mm was chosen as the minimal possible distance between the nozzle and the sample that did not result in significant liquid surface disturbance by the feed gas flow. Virtually identical amounts of H_2_O were measured in the D_2_O sample at the two distances (Table 1, entries 7, 8 and 5, 6).

These results clearly demonstrate that both H_2_O and D_2_O are delivered into the liquid sample with equal efficiency at distances of both 10 and 4 mm from the jet nozzle to the liquid sample, which allows direct use of these conditions in the investigation of the origin of species induced in the liquid sample by plasma.

### Reactive species in the liquid sample

#### Hydrogen peroxide

H_2_O_2_ is considered to be a key component in the wound‐ healing and anti‐microbial and anti‐cancer properties of cold plasma.^8^, ^37^ A multitude of possible reactions can lead to the formation of H_2_O_2_ 7, 26, 27, 28 from water vapour and oxygen; the most straightforward pathway is shown in Equations ((1))–((2)). The effects of humidity and the oxygen admixture in the feed gas on the H_2_O_2_ content in the plasma‐treated liquid were investigated.

(1) $$H{}_{2}O\to \cdot \mathrm{OH}+\cdot H$$

(2) $$\cdot \mathrm{OH}+\cdot \mathrm{OH}\to H{}_{2}O{}_{2}$$

(3) ·H+O→·OH

**Figure 2:**
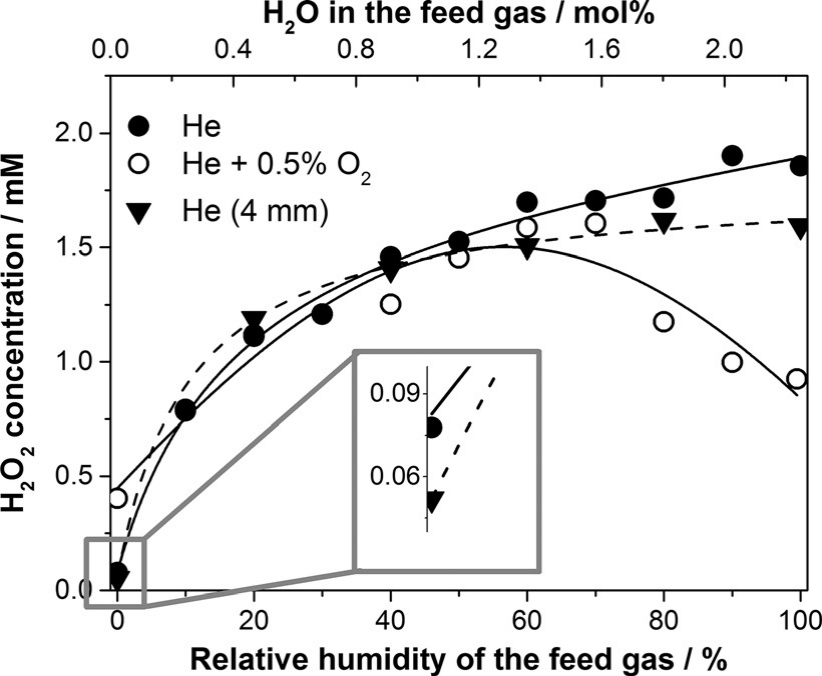
H_2_O_2_ concentration in a liquid sample as a function of feed gas humidity: helium (•) and helium with 0.5 % O_2_ (○) at 10 mm distance from the nozzle to the sample and helium at 4 mm (▼) distance from the nozzle to the sample. In this figure the lines are added to guide the eye.

The concentration of H_2_O_2_ in the liquid sample was measured by UV/Vis spectroscopy using a solution of potassium oxotitanate dihydrate in H_2_O/H_2_SO_4_.^38^ The evaporation of water from the liquid sample was included in the calculation of the final H_2_O_2_ concentration. As the H_2_O_2_ vapour pressure was at least 10 times lower than that of H_2_O under all experimental conditions,^39^, ^40^ the evaporation of H_2_O_2_ was disregarded. The results are presented in Figure 2 and show that in the absence of H_2_O vapour in the feed gas, only a minor amount of H_2_O_2_ is detected in the liquid sample. The concentration of H_2_O_2_, however, increases dramatically with increased feed gas humidity. This observation suggests that H_2_O_2_ is not formed in the liquid by dissociation of H_2_O [Eq. (1) and (2); this would not show such a strong dependence on the feed gas humidity], but instead is formed in the gas phase and then diffuses into the liquid sample.

The plot of H_2_O_2_ concentration (Figure 2) flattens out at high humidity. This is attributed to a reduced electron density in the plasma with increased molecular content.^41^ A similar effect was observed on introducing an oxygen admixture (0.5 %) into the feed gas (Figure 2), however, in this case the amount of H_2_O_2_ in the liquid decreased at elevated humidity (>70 %) of the feed gas. Here, in addition to a drop in the electron density of the plasma, the amount of H_2_O_2_ could also be reduced by side‐reactions involving some of the species that form H_2_O_2_ [e.g., Eq. ((4))] or by secondary reactions, for example, the peroxone process.^42^ Varying the O_2_ admixture at low humidity, however, did not result in significant changes in the H_2_O_2_ concentration in the liquid sample (see Figure S6 in the Supporting Information).

(4) $$\cdot \mathrm{OH}+O{}_{3}\to \cdot \mathrm{OOH}+O{}_{2}$$

To test the origin of the minor amount of H_2_O_2_ observed with a dry feed gas, the distance between the nozzle and the sample was decreased to 4 mm. This short distance significantly reduces the interaction of the plasma jet with the wet ambient gas inside the reactor (the gas inside the reactor contains evaporated water). The diffusion of species from the ambient gas into the plasma effluent was demonstrated by performing an experiment in which the ⋅NO radical was detected with the (MGD)_2_Fe^2+^ (MGD=*N*‐methyl‐d‐glucaminedithiocarbamate) spin trap in plasma with air as the ambient gas and helium as the feed gas. The amount of trapped ⋅NO reduced drastically at 4 mm compared with at 10 mm distance from the nozzle to the sample (data not shown).

The experimental data revealed that the amount of H_2_O_2_ formed with dry helium at 4 mm distance was somewhat lower than at 10 mm, which supports the hypothesis that this minor amount was formed from the ambient humidity in the reactor (see expanded region in Figure 2). This amount increased with the addition of oxygen, possibly due to reactions of atomic oxygen [e.g., Eq. (3)].

The amount of H_2_O_2_ detected with a wet feed gas was almost the same for distances of 10 and 4 mm between the nozzle and the sample (Figure 2) and hence was not affected by the interaction of the plasma jet with the wet gas in the reactor. As the delivery of the species from the gas phase into the liquid sample was nearly equal at these distances (Table 1) and strongly dependent on the feed gas humidity, we propose that the H_2_O_2_ induced in the liquid is largely formed from the species generated inside the plasma tube (but not in the region below the nozzle of the plasma tube as observed for the ⋅OH and ⋅H radicals, see below) and subsequently delivered into the liquid sample.

#### ⋅OH radical

The biological effects of cold plasma treatment are often attributed to the formation of hydroxyl radicals,^9^, ^43^ an important precursor of hydrogen peroxide [see Eq. (2)]. The hydroxyl radical is a very short‐lived species and in most cases cannot be detected in liquids directly.^44^ In this work, spin trapping in conjunction with EPR spectroscopy was used to detect these reactive radicals.

**Figure 3:**
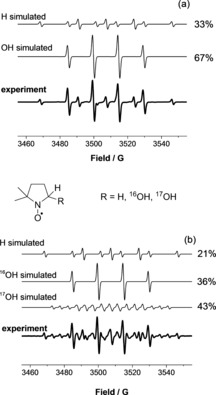
Typical experimental and simulated EPR spectra of DMPO radical adducts formed in plasma‐exposed aqueous solutions of DMPO in (a) H_2_O and (b) H_2_^17^O. DMPO–H: *a*_N_=16.4 G, *a*_H_=22.6 G (×2); DMPO–OH: *a*_N_=15.0 G, *a*_H_=14.7 G; DMPO–^17^OH: *a*_N_=14.9 G, *a*_H_=14.8 G, *a*_17O_=4.7 G.

The concentration of the ⋅OH radical in liquid samples was assessed by exposing aqueous solutions of the DMPO spin trap to plasma. The subsequent EPR analysis of the solutions revealed the trapping of both ⋅OH and ⋅H radicals (Figure 3); the DMPO–OH radical adduct in the liquid sample was observed in concentrations of up to around 23.5 μm, whereas the concentration of DMPO–H in most cases did not exceed 2.3 μm (see Figure S7 in the Supporting Information).

**Figure 4:**
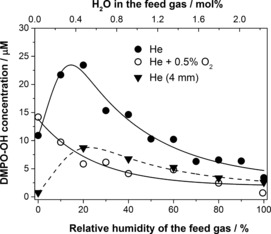
DMPO–OH adduct concentration in plasma‐treated liquid samples as a function of feed gas humidity: helium (•) and helium with 0.5 % O_2_ (○) at 10 mm distance from the nozzle to the sample and helium at 4 mm (▼) distance from the nozzle to the sample.

The concentration profile of the DMPO–OH adduct in plasma‐treated water is shown in Figure 4. Although these concentrations do not represent the exact amount of ⋅OH radical generated by the plasma due to side‐reactions and the limited selectivity of DMPO as a spin trap for ⋅OH, the changes in relative concentrations of the DMPO–OH adduct match those of the ⋅OH radical. The concentration of the DMPO–OH adduct in the liquid sample was significantly affected by feed gas humidity. In particular, the concentration of the adduct detected at 4 mm distance between the sample and the nozzle was much lower for dry helium than for wet helium. This observation strongly suggests that the trapped ⋅OH radical was not formed in the liquid, as in this case its concentration in the liquid would have been highest with the dry feed gas. The decrease in DMPO–OH concentration with increased humidity, and an even more rapid decay for oxygen‐containing feed gas (Figure 4; see also Figure S8 in the Supporting Information) can be attributed to a reduced electron density of the plasma with increasing molecular content.

The very significant difference between the DMPO–OH adduct concentrations for samples treated at distances of 4 and 10 mm (Figure 4) suggests that the concentration of ⋅OH in the liquid sample is strongly affected by the interaction of the plasma jet with the wet gas inside the reactor. This is in contrast to the trends observed for H_2_O_2_. We conclude therefore that whereas the H_2_O_2_ delivered into the liquid is generated inside the plasma tube, the ⋅OH radical in the liquid phase originates from the plasma jet, that is, the region below the nozzle of the quartz tube.

To further confirm that the hydroxyl radicals are formed in the gas phase and not in the liquid, isotopically labelled water (H_2_^17^O) was employed. This made it possible to differentiate between the ⋅OH radicals created in the liquid phase and those formed in the gas phase and subsequently delivered into the liquid sample. Samples of DMPO in H_2_^17^O were exposed to helium plasma, dry and with 10 % humidity (H_2_^16^O; Table 2). DMPO–^16^OH and DMPO–^17^OH adducts have distinct EPR signals (Figure 3), and the concentration of each species can hence be independently determined.

**Table 2:** Concentration of DMPO–^16^OH and DMPO–^17^OH radical adducts in a liquid H_2_^17^O sample after 60 s of plasma exposure.

Entry	Relative humidity (H_2_^16^O)	Distance^[a]^	Adduct concentration [μm]	
	of the feed gas [%]	[mm]	⋅^17^OH	⋅^16^OH
1	0^[b]^	10	5.6	4.8
2	10	10	8.3	18.3
3	10	4	2.9	9.1

[a] Distance from the nozzle to the sample. [b] Feed gas contained trace amounts of water vapour.

With the dry feed gas, the only major source of ⋅OH radicals is the sample (either liquid or evaporated sample), but with the wet feed gas, the ⋅OH radicals could come from either the sample or the feed gas. The introduction of H_2_O vapour into the feed gas makes it possible to study the origin of the ⋅OH radicals and the effect of mixing the feed gas with the wet gas inside the reactor. For example, let us consider an experiment with a DMPO solution in liquid H_2_^17^O and H_2_^16^O vapour in the feed gas. In this case, ⋅^16^OH radicals (if any) could only originate from the gas phase and ⋅^17^OH radicals could be formed in either the liquid phase or the atmosphere in the reactor which contains evaporated H_2_^17^O.

An additional factor, however, must be taken into account. The composition of the liquid phase changes during plasma treatment due to sample evaporation and condensation of water vapour from the feed gas. The composition of the liquid sample (H_2_^16^O to H_2_^17^O ratio) was determined by studying the hydrolysis of cinnamoyl chloride by water. The reaction product, cinnamic acid, was analysed by HRMS to obtain the ratio of cinnamic acid molecules with ^16^O and ^17^O isotopes, that is, those hydrolysed by H_2_^16^O and H_2_^17^O, respectively. The H_2_^16^O content in the plasma‐treated liquid H_2_^17^O sample was in all cases low (see Table S4 in the Supporting Information).

With the dry helium feed gas, the relative amount of the DMPO–^16^OH radical adduct was quite significant, around 45 % (Table 2, entry 1). This DMPO–^16^OH adduct here probably originates from H_2_^16^O impurity in the helium feed gas and the H_2_^16^O content of the liquid sample (ca. 18 %, see Table S4 in the Supporting Information). This result alone strongly suggests that the ⋅OH radical is not formed in the liquid phase (in this case over 80 % of the DMPO–^17^OH adduct would have been formed). When a small amount of water vapour was introduced into the feed gas, the relative amount of DMPO–^16^OH radical adduct increased to 70 % (Table 2, entry 2), even though only a minor increase in H_2_^16^O content was observed in the liquid phase (see Table S4). This, again, is consistent with ⋅OH radical formation in the gas phase. At a distance of 4 mm (which results in reduced interaction of plasma jet with the wet gas inside the reactor, see above), the concentrations of both DMPO–OH adducts decreased, with the DMPO–^17^OH adduct most affected (entry 3), in good agreement with the proposed formation of the ⋅OH radical in the gas phase, in the region below the nozzle of the plasma tube.

#### ⋅H radical

Similarly to the investigations of H_2_O vapour delivery (Table 1) and isotopically labelled ⋅OH radicals (Table 2), the use of hydrogen isotopes made it possible to differentiate between the ⋅H radicals created in the liquid phase and those that were delivered into the liquid sample from the gas phase.

**Figure 5:**
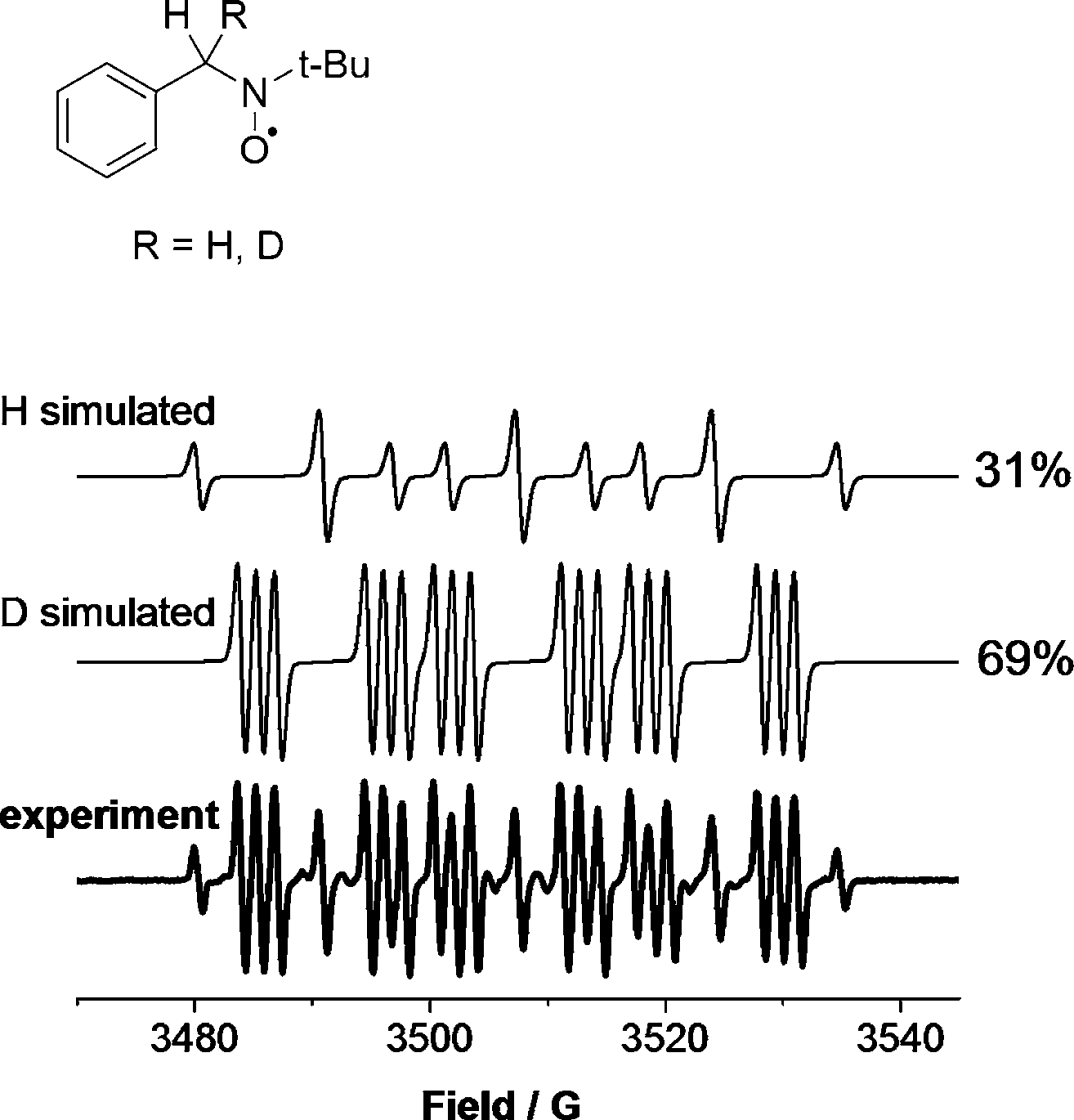
Typical experimental and simulated EPR spectra of PBN radical adducts formed in plasma‐treated H_2_O or D_2_O solutions. PBN–H: *a*_N_=16.6 G, *a*_H_=10.8 G (×2); PBN–D: *a*_N_=16.7 G, *a*_H_=10.8 G, *a*_D_=1.6 G.

The PBN spin trap in either D_2_O or H_2_O was treated with plasma using a feed gas with admixtures of either H_2_O or D_2_O. Different spin traps have different affinities towards a certain group of radicals; the treatment of *N*‐*tert*‐butyl‐α‐phenylnitrone (PBN) solutions with an oxygen‐free plasma led to the predominant formation of PBN–H (or D) adducts, in contrast to the results obtained with DMPO, for which, under most conditions, the DMPO–OH adduct was formed predominantly. The EPR spectrum of a typical PBN–H and PBN–D radical adduct mixture is shown in Figure 5.

Here, similarly to the H_2_^17^O experiments, changes in the composition of the liquid sample due to sample evaporation and feed gas condensation must be considered. This was accounted for by using the data in Table 1. Additionally, the rates of cleavage of the O−H and O−D bonds in H_2_O and D_2_O, respectively, are different due to the primary kinetic isotope effect (KIE). The KIE would thus lead to potentially different concentrations of ⋅H and ⋅D radicals under otherwise identical conditions and hence must be taken into account. The KIE in our system was estimated by the following method. Liquid samples containing different ratios of H_2_O/D_2_O and a PBN spin trap were treated with plasma using a feed gas that was fully saturated with vapour of the same composition. The apparent KIE has a value of 3.0, as calculated from the ratio of the PBN–H and PBN–D adducts (see Table S5 and related discussion in the Supporting Information). This value is in the range typical of KIEs for H/D systems. Our method for KIE estimation is valid regardless of whether the ⋅H/⋅D radicals are formed in the liquid sample or in the feed gas.

The results of experiments with different isotopes in the gas and liquid phases show that a minor amount of the PBN–H radical adduct was formed in the absence of H_2_O in both the feed gas and liquid (Table 3, entries 1 and 5). The amount of PBN–H adduct formed was higher at 4 mm distance than at 10 mm distance. Similar results were obtained by using the DMPO spin trap (see Figure S7 in the Supporting Information). Therefore, we conclude that this ⋅H adduct originates from impurities in the feed gas (e.g., H_2_). The total radical concentration decreased with increased feed gas humidity (Table 3; see also Table S6 in the Supporting Information). This can be explained by a decreased electron density under these conditions, similar to the results obtained with the ⋅OH radical.

**Table 3:** Concentrations of PBN–H and PBN–D radical adducts after plasma exposure with H_2_O and D_2_O in the feed gas and the liquid sample.

Entry	Plasma exposure conditions		Adduct concentration^[b]^ [μm]	
	Distance^[a]^ [mm]	Relative humidity [%]	⋅H	⋅D

**D_2_O liquid sample/H_2_O vapour in the feed gas**				

1	10	–	1.9	9.3
2		10	8.1	2.3
3		50	3.6	0.3
4		100	3.2	0.2
				
5	4	–	4.3	6.6
6		10	7.4	1.2
7		50	4.1	0.2
8		100	3.1	0.2

**H_2_O liquid sample/D_2_O vapour in the feed gas**				

9	10	–	12.9	0
10		10	9.2	6.2
11		50	3.9	7.5
12		100	1.3	6.2
				
13	4	–	13.6	0
14		10	4.3	8.5
15		50	2.5	16.3
16		100	1.1	6

[a] Distance from the nozzle to the sample. [b] Additional PBN adducts, for example, PBN–OH were also detected (data not shown).

The data in Table 3 clearly show that the trapped ⋅H radical does not originate from the liquid phase. The clearest evidence for this comes from entry 12, which reports the results of plasma treatment of H_2_O by using D_2_O‐saturated feed gas. At the end of the plasma exposure, the liquid phase is still dominated by H_2_O (the concentration of D_2_O is below 15 %). At the same time, little ⋅H is trapped; over 80 % ⋅D is trapped. If one takes into account the KIE and the fact that some trapped ⋅H comes from impurities in the feed gas, it becomes clear that only a very small amount (if any) of trapped ⋅H/⋅D radical originates in the liquid phase. Additional calculations to support this are found in Table S7 and the related discussion in the Supporting Information.

Entries 1, 5, 9 and 13 in Table 3 also show that most of the trapped ⋅H/⋅D does not originate in the quartz tube. These experiments were carried out with dry helium gas. A significant amount of trapped ⋅D (entries 1 and 5) suggests that it originates from the evaporated liquid, for example, in the plasma jet mixed with the atmosphere in the reactor (which contains evaporated D_2_O), just like the ⋅OH radical. This conclusion is further supported by the high concentration of the ⋅H adduct (entries 9 and 13) observed with dry helium feed gas and a H_2_O liquid sample.

Comparison of entries 1 and 5 of Table 3 leads to an unexpected conclusion. In this experiment, a D_2_O sample was treated with H_2_O‐saturated plasma and hence the⋅D radical must originate from the evaporated sample. Surprisingly, however, only a moderate reduction in the trapped ⋅D was observed at 4 mm distance between the nozzle and the sample as compared with the 10 mm distance. This is very different from the ⋅OH radical (which also originates from the plasma effluent and has a greatly reduced concentration at 4 mm, see Figure 4). We infer therefore that trapping of the ⋅H radical is less affected by the distance between the nozzle and the sample, possibly because it is formed closer to the plasma/liquid interface.

#### Superoxide O_2_⋅^−^

Another important radical formed in oxygen‐containing plasmas is the superoxide radical anion O_2_⋅^−^.^20^, ^45^ It can be formed from the reaction of molecular oxygen with electrons or by the deprotonation of the hydroperoxyl radical ⋅OOH, which can be produced, for example, by the reaction of ozone with the hydroxyl radical [Eq. (4)].^7^, ^26^ Other pathways leading to the formation of the superoxide radical include secondary post‐exposure reactions (e.g., the peroxone process^42^).

**Figure 6:**
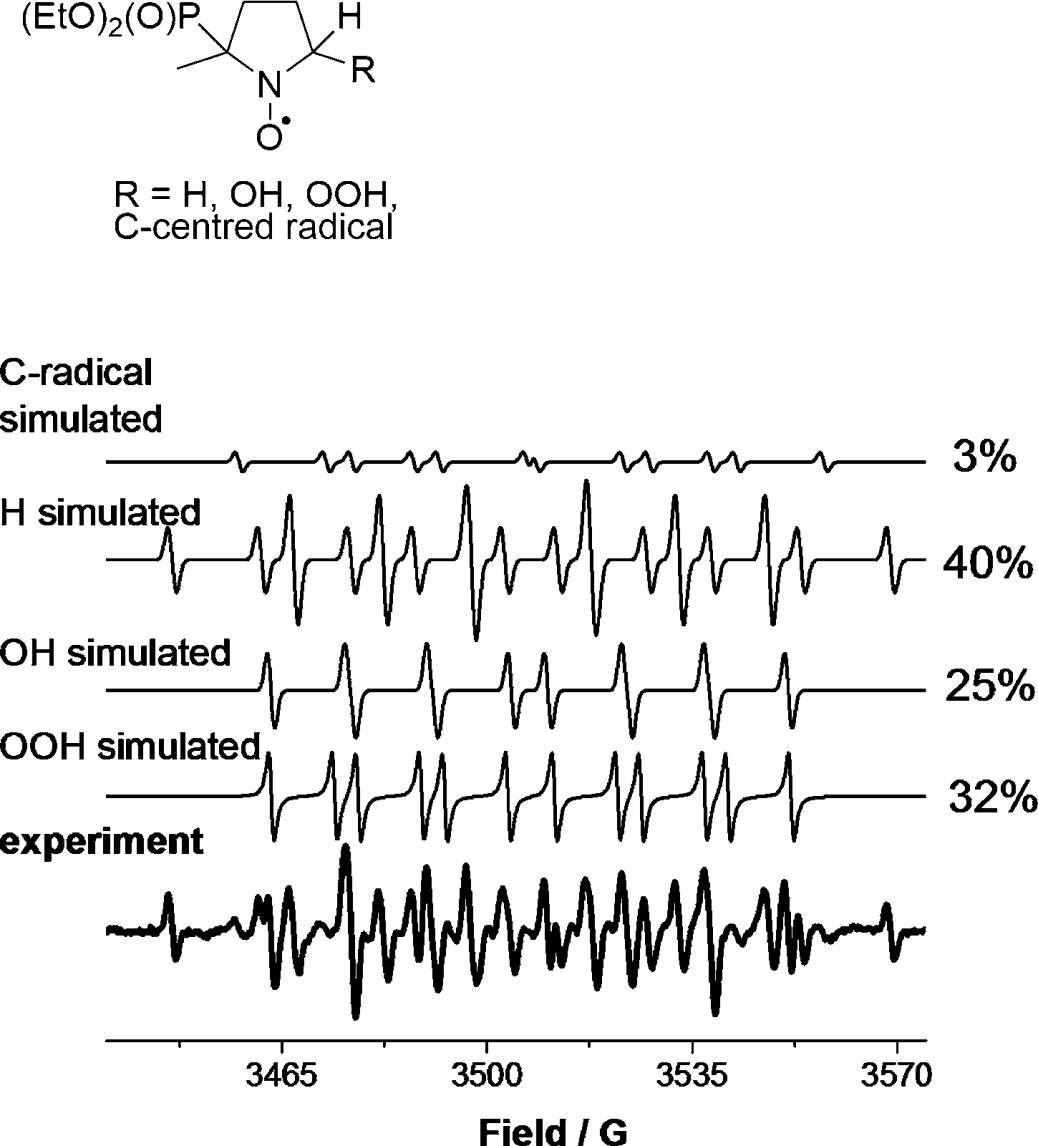
Typical experimental and simulated EPR spectra of DEPMPO radical adducts formed in plasma‐treated aqueous solutions. DEPMPO–OOH (conformer 1): *a*_N_=14.0 G, *a*_H_=13.1 G, *a*_P_=47.3 G; DEPMPO–OOH (conformer 2): *a*_N_=12.0 G, *a*_H_=9.7 G, *a*_P_=48.7 G; DEPMPO–OH: *a*_N_=14.0 G, *a*_H_=13.0 G, *a*_P_=47.2 G; DEPMPO–H: *a*_N_=15.3 G, *a*_H_=20.7 G (×2), *a*_P_=50.5 G; DEPMPO adduct of C‐centred radical: *a*_N_=14.9 G, *a*_H_=19.3 G, *a*_P_=50.7 G.

The 5‐(diethoxyphosphoryl)‐5‐methyl‐1‐pyrroline *N*‐oxide (DEPMPO) spin trap was employed to detect the O_2_⋅^−^ radical; the DEPMPO–OOH radical adduct thus formed is much more stable than the DMPO–OOH adduct. The EPR analysis of plasma‐treated DEPMPO (Figure 6) showed the formation of spin adducts with ⋅H, ⋅OH and O_2_⋅^−^ radicals (calculated as the sum of two conformers) in most experiments. Additionally, carbon‐centred radical was observed (the exact structure of the adduct was not determined; its simulated hyperfine values match literature values for various carbon‐centred radical adducts of DEPMPO^46^). The carbon‐centred adduct is likely a degradation product of DEPMPO: it was not observed in the presence of molecular oxygen in the feed gas (see Figure S11 in the Supporting Information), consistent with the high reactivity of carbon‐centred radicals with oxygen.^47^ In any case, the amount of this adduct was substantially lower than those of the other observed species, such as the hydroxyl and superoxide radical adducts (see Figures S9–S11).

**Figure 7:**
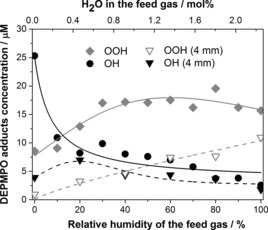
DEPMPO–OOH (♦ and ▿) and DEPMPO–OH (• and ▼) adduct concentrations in the liquid at distances of 10 and 4 mm from the nozzle to the sample as a function of feed gas humidity.

The results of the spin‐trapping experiments with DEPMPO at distances of 10 and 4 mm from the nozzle to the sample are presented in Figure 7. The amounts of both the DEPMPO–OH and DEPMPO–OOH radical adducts decreased when the experiments were performed at 4 mm distance, which suggests that the O_2_⋅^−^ radical was largely formed in the gas phase inside the reactor, similarly to ⋅OH (as was also demonstrated for the DMPO spin trap, see above).

It is worth noting that the relative amounts of both the ⋅OH and O_2_⋅^−^ adducts depend on the feed gas humidity. For instance, DEPMPO–OH is the dominant adduct with the dry feed gas, whereas DEPMPO–OOH dominates at high feed gas humidity. This suggests that not just the amount, but also the selectivity of radical generation can be controlled by different plasma conditions, such as plasma feed gas humidity and oxygen content (see Figure S12 in the Supporting Information). Such an ability to tune the nature of the reactive species in the liquid sample presents great potential for possible cold plasma applications.^48^

#### O_3_/^1^O_2_/O

Ozone and ^1^O_2_ are readily available in oxygen‐rich plasma systems.^34^, ^49^, ^50^ These species may lead to the formation of other reactive species, for example, ⋅OH and O_2_⋅^−^ radicals, by post‐exposure reactions in the liquid sample. Thus, we assessed the concentrations of ozone, atomic oxygen and ^1^O_2_ in the liquid sample.

Although several methods for measuring the concentration of ozone and ^1^O_2_ in liquids have been reported in the literature, many are not selective in the case of plasma‐treated liquids. In a recent report, Kohno and co‐workers determined the oxidising species in solutions by the oxidation of 2,2,5,5‐tetramethyl‐3‐pyrroline‐3‐carboxamide (TPC) to form a stable radical which was analysed by EPR spectroscopy. The authors were able to estimate the concentration of singlet delta oxygen ^1^O_2_ by employing sodium azide, which acts as a selective scavenger for ^1^O_2_.^21^, ^51^

In the present study, 60 mm aqueous solutions of 2,2,6,6‐tetramethylpiperidine (TEMP)^52^ with and without the addition of NaN_3_ were exposed to plasma and the concentration of the oxidising species was estimated from the intensity of the EPR signal of TEMPO. Control experiments with H_2_O_2_ and superoxide (added as KO_2_), separately and combined, showed that these compounds do not produce TEMPO at concentrations up to 60 mm and hence do not contribute to the observed signal. Ozone, on the other hand, did produce TEMPO. The reactivity of atomic oxygen with TEMP is unknown but it is reasonable to assume that it contributes to the formation of TEMPO.

The data obtained in the preliminary experiments revealed that the concentrations of trapped O_3_/^1^O_2_/O decreased dramatically in all cases when water vapour was introduced into the feed gas. For instance, a TEMP solution treated with plasma containing 0.5 % oxygen in helium and a humidity above 20 % yielded TEMPO at a concentration below 15 μm, whereas in the case of the dry feed gas the concentration at which TEMPO was produced increased to around 70 μm (see Figure S13 in the Supporting Information). This is in agreement with the results reported by Reuter and co‐workers, who observed that the amount of ozone in the gas phase decreased substantially when water vapour was introduced into the feed gas even at low levels.^34^ This is most likely due to the decay of formed ozone (water is an extremely effective quencher of ozone, producing, for example, the hydroxyl radical and molecular oxygen).

**Figure 8:**
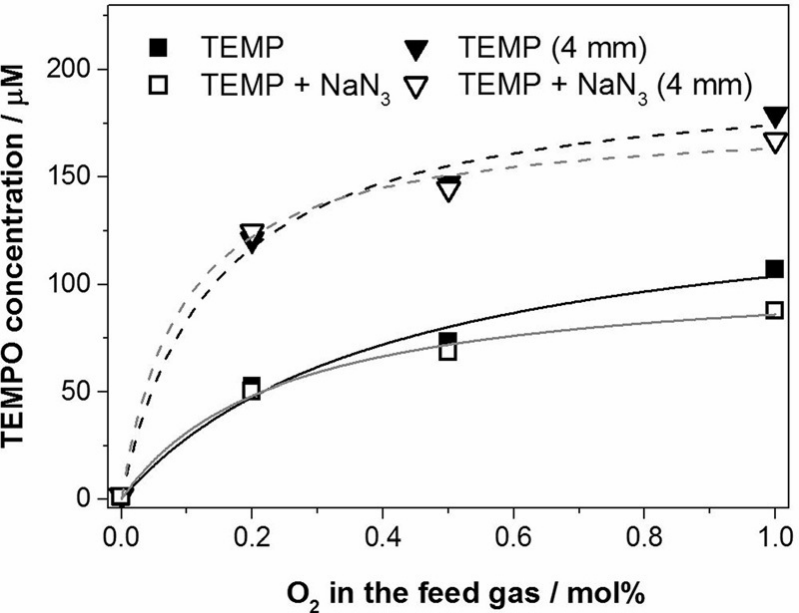
TEMPO concentration in plasma‐treated aqueous solutions of TEMP (▪ and ▼) and TEMP with added sodium azide and (□ and ▿) at the 10 and 4 mm distance from the nozzle to the sample, as a function of the O_2_ admixture in the dry feed gas.

Only negligible amounts of TEMPO were formed in the absence of added molecular oxygen (see Figure S13 in the Supporting Information). The amount of TEMPO increased somewhat when humidity was introduced into the dry feed gas. This suggests that small amounts of O_3_/^1^O_2_/O can be formed from water molecules. The concentration of TEMPO, however, increased dramatically with an increase in the oxygen admixture in the feed gas (Figure 8). This unambiguously demonstrates that the bulk of O_3_/^1^O_2_/O originates from the molecular oxygen added to the feed gas.

At 4 mm distance from the nozzle to the sample, the concentration of TEMPO increased approximately two‐fold for all oxygen concentrations in the feed gas. The decreased concentrations of O_3_/^1^O_2_/O at the longer distance (10 mm) can be tentatively attributed to their reactions with ⋅OH and other species present in the plasma jet mixed with the evaporated liquid sample.

The addition of ^1^O_2_ scavenger NaN_3_ did not significantly affect the concentration of TEMPO (Figure 8). Similar results were obtained when the feed gas contained 20 % water vapour (see Figure S14 in the Supporting Information). This indicates that the contribution of ^1^O_2_ to the oxidation of TEMPO is negligible in our investigation, and the data in Figure 8 are largely attributed to O_3_/O in the liquid sample.

## Conclusion

The treatment of aqueous samples with non‐thermal atmospheric pressure plasma jets results in the generation of a number of reactive species. This work was aimed at gaining an understanding of where these compounds originate from, and whether experimental parameters (such as the feed gas composition and the distance between the nozzle and the sample) have the potential to tune their concentrations. A combination of spin‐trapping/EPR spectroscopy and conventional analytical methods made it possible to assess the relative concentrations of H_2_O_2_, ⋅OH, O_2_⋅^−^, ⋅H, ^1^O_2_ and O_3_/O in solutions treated with a parallel field kHz driven atmospheric pressure plasma jet. The ambient atmosphere was controlled by means of an in‐house built reactor.

For the first time, the possibility of experimentally distinguishing between reactive species generated from the liquid sample and the feed gas has been demonstrated. This was achieved by 1) specific labelling of one phase with hydrogen or oxygen isotopes (i.e., D_2_O or H_2_^17^O) and 2) variation of the distance between the plasma jet nozzle and the sample (the interaction of the plasma with the evaporated liquid is significantly reduced at short distances between the nozzle and the sample). This approach allowed us to perform such an analysis with various plasma jets operated under different conditions.

The results show that different reactive species detected in the plasma‐treated liquid sample originate in different regions of the plasma interaction setup. H_2_O_2_ delivered to the sample is almost exclusively created from species in the plasma tube. On the other hand, ⋅H, ⋅OH and superoxide radicals originate in the plasma effluent, that is, in the volume between the plasma nozzle and the sample in which some interaction of the plasma jet with the evaporated sample takes place. Different radicals, however, show different trends, with ⋅H radicals observed even at short distances between the nozzle and the sample; it was hypothesised that ⋅H originates in the volume close to the plasma/liquid interface.

The data obtained in this study make it possible to rationally design certain plasma treatment conditions. For instance, we found that O_3_/^1^O_2_/O are only delivered into the liquid sample if O_2_ is present in the feed gas (e.g., only a negligible amount of these species can be formed from water molecules). In another example, variation of the feed gas composition significantly changes the relative amounts of ⋅OH and O_2_⋅^−^ radicals trapped in the liquid phase.

## Supporting information

As a service to our authors and readers, this journal provides supporting information supplied by the authors. Such materials are peer reviewed and may be re‐organized for online delivery, but are not copy‐edited or typeset. Technical support issues arising from supporting information (other than missing files) should be addressed to the authors.

Supplementary
