# Supplementary material for: Non‐Thermal Plasma in Contact with Water: The Origin of Species
Source: Chemistry. 2016 Feb 2;22(10):3496–505. doi: 10.1002/chem.201503771 (PMC4797710; doi:10.1002/chem.201503771)
Supplement: Supplementary file 1 — Supplementary [file CHEM-22-3496-s001.pdf]

CHEMISTRY
A European Journal

Supporting Information

Non-Thermal Plasma in Contact with Water: The Origin of Species

Yury Gorbanev,[a, b] Deborah O'Connell,*[b] and Victor Chechik*[a]

chem_201503771_sm_miscellaneous_information.pdf

Supporting information

Raw data is available at: DOI 10.15124/15f674be-e9ca-4a00-9ba6-3c24e70a6aa4

Table of contents

Experimental details 2
Figure S1. Voltage and return current waveforms with different feed gas compositions 4
Table S1. The results of the OES analysis of the gas phase plasma inside the quartz tube between the electrodes 4
Figure S2. Calibration curves for the analyses 5
Table S2. Error assessment 6
Figure S3. A schematic representation of the experimental setup 6
Table S3. H_2O concentration in the feed gas with helium passing through a Drechsel flask 6
Table S4. H_2O content in the liquid $H_2{}^{17}O$ sample after plasma exposure 7
Figure S4. EPR spectra of DMPO and PBN spin adducts in experiments with magnesium fluoride window 7
Figure S5. H_2O amount in a D_2O liquid sample delivered by helium flow 7
Figure S6. H_2O_2 concentration in the liquid as a function of O_2 content of the feed gas 8
Figure S7. DMPO-H adduct concentration in the liquid sample 8
Figure S8. DMPO-OH adduct concentration in the liquid 8
Table S5. Experimental investigation of primary kinetic isotope effect 9
Table S6. Concentrations and relative amounts of PBN-H and PBN-D radical adducts 10
Table S7. Experimental and calculated relative amount of PBN adducts 11
Figure S9. DEPMPO-OOH and DEPMPO-OH radical adduct concentration 11
Figure S10. The concentration of the DEPMPO-H adduct 12
Figure S11. The concentration of the DEPMPO adduct of a carbon-centred radical 12
Figure S12. DEPMPO-OOH and DEPMPO-OH adduct concentrations 13
Figure S13. Concentration of the formed TEMPO 14
Figure S14. Concentration of the formed TEMPO with added sodium azide 14

Experimental

Methods

The plasma was ignited in a quartz tube (4 mm ID and 6 mm OD, 100 mm length) surrounded by copper electrodes (10 mm width) separated by 20 mm. A PVM500 Plasma Resonant and Dielectric Barrier Corona Driver power supply (Information Unlimited) was used to sustain the plasma. A high voltage probe (Tektronix P6015A) and current probe (Ion Physics Corporation CM-100-L) were used with a Teledyne LeCroy WaveJet 354A oscilloscope to measure time resolved current and voltage. Voltage and frequency were kept constant throughout all experiments at 18.3 ± 0.2 kV (peak-to-peak) and 24.9 kHz, respectively. The return current values were between *ca.* 4 and 7 mA. The voltage and current waveforms at some of the experimental conditions are shown in Fig. S1. OES measurements of the plasma between the electrodes were performed with Ocean Optics HR-4000CG-UV-NIR spectrophotometer (Table S1).

The plasma was operated with a feed gas of helium with oxygen and water admixtures controlled by mass flow controllers (MFCs) (Brooks Instruments and Brooks Instruments 0254 microcomputer controller). All experiments were carried out with a total flow of feed gas of 2 L/min. The percentage of the O_2 admixture is shown in vol%, and the concentration of water vapour is quoted in percent of the saturation and mol%.

The experimental setup was positioned inside a large Faraday cage with the mesh size of 22 mm.

Materials

Hydrogen peroxide H_2O_2 (30%), sulphuric acid H_2SO_4 (>95%) and sodium azide NaN_3 (≥99.5%) were purchased from Fluka. Deuterium oxide D_2O (99.9 atom% D), *N-tert*-butyl-*α*-phenylnitrone (PBN) (98%), 2,2,6,6-tetramethylpiperidine (TEMP) (≥99%), 2,2,6,6-tetramethylpiperidine 1-oxyl (TEMPO) (98%), sodium *p*-toluenesulfonate (sodium tosylate) (95%), cinnamoyl chloride (98%) and $H_2{}^{18}O$ (97%) were obtained from Sigma-Aldrich. 5,5-Dimethyl-1-pyrroline *N*-oxide (DMPO) (≥99%) and 5-diethoxyphosphoryl-5-methyl-1-pyrroline *N*-oxide (DEPMPO) (≥99%) were purchased from Dojindo Molecular Technologies, Inc. and Enzo Life Sciences, respectively. Potassium bis(oxalato)oxotitanate(IV) dihydrate was obtained from Alfa Aesar. $H_2{}^{17}O$ was purchased from Icon Isotopes. De-ionised water was used for the preparation of the solutions. Helium He (A Grade, 99.996%) and oxygen O_2 (Zero Grade, 99.6%) were supplied by BOC UK. All chemicals were used as received.

Analysis

Electron paramagnetic resonance (EPR) measurements were carried out on a Bruker EMX Micro EPR spectrometer. The EPR analysis parameters were as follows: frequency 9.83 GHz, power 3.17 mW, modulation frequency 100 kHz, modulation amplitude 1 G, time constant 40.96 msec, number of scans 5, sweep width 100 G (DMPO and PBN adducts, TEMPO) or 170 G (DEPMPO addcuts). For the measurements, all samples were contained in glass capillary tubes (80 x 1 mm) purchased from Marienfeld Laboratory Glassware. EPR calibration was performed using aqueous solutions of a stable radical (TEMPO) in a range of concentrations 2-200 μM (Fig. S2).

After each plasma exposure experiment, the samples were immediately placed in a capillary tube. The overall time between the exposure and recording the spectrum was 2 minutes.

EPR spectra simulations were performed on NIH P.E.S.T. WinSIM software ver. 0.96 [available online at http://www.niehs.nih.gov/research/resources/software/tox-pharm/tools/].

Concentration of H_2O_2 in the samples was determined by UV-Vis measurements performed on a UV-1800 Shimadzu UV-Vis Spectrophotometer with Optical Glass High Precision Cells (10 mm light path) provided by Hellma Analytics. UV-Vis calibration was done using 500 μL titanium(IV) reagent with added 300 μL aqueous hydrogen peroxide solutions in a range of concentrations 0.0979-4.895 mM (Fig. S2). Titanium(IV) reagent was prepared by dissolving 3.54 g of potassium bis(oxalato)oxotitanate(IV) dihydrate in a mixture of 27.2 mL of sulphuric acid and 30 mL of H_2O, and diluting the resulting solution to a total volume of 100 mL.

UV-Vis spectra of samples were recorded by adding a mixture of 65 μL of plasma-exposed sample (taken immediately after plasma exposure) with 235 μL of H_2O to 500 μL of titanium(IV) reagent. The resulting solutions were incubated for 1 min before analysis. The H_2O_2 concentration was determined from the UV-Vis intensity of the peak at 400 nm.

For ^1H NMR analysis, 50 μL of plasma-exposed sample was added to 500-600 μL of 0.5 M sodium tosylate solution in D_2O in a Young NMR tube and kept under argon. The NMR spectra were recorded on a JEOL ECS400 spectrometer.

The composition of $H_2{}^{17}O$ samples was analysed as follows. In a typical experiment, 10 μL of a 1.5 M solution of cinnamoyl chloride in acetonitrile was added to 10-15 uL of samples containing $H_2{}^{17}O$ in a small vial filled with argon. The mixture was heated to 60 °C for 2 min to allow full hydrolysis. The samples were cooled down and diluted to *ca.* 100 μM concentrations with 1:1 water:acetonitirile mixture. The cinnamic acid formed as a result of the cinnamoyl chloride hydrolysis was analysed using high-resolution MS spectrometry. Mass spectra were acquired using a Bruker 9.4T solariX XR Fourier transform ion cyclotron resonance (FT-ICR) mass spectrometer (Bremen, Germany). The samples were ionized in positive ion mode using the ESI ion source. Spectra were measured with a transient length of 0.84 s

resulting in a resolving power of 400000 at *m/z* 150. The instrument was externally calibrated using sodium formate clusters. The percentage of H_2O in a $H_2{}^{17}O$ sample was calculated from relative signal ratio of cinnamic acid containing ^{16}O and ^{17}O isotopes (m/z 149 (^{16}O) and 150 (^{17}O)). To correct the obtained values with regard to the contribution of cinnamic acid present in the pristine acetonitrile solution, a calibration of the solution was performed with $H_2{}^{18}O$ samples with different amount of added H_2O (m/z 149 (^{16}O) and 151 (^{18}O) (Fig. S2).

The concentrations of all reactive species in the liquid samples are quoted after correction for the material lost through solvent evaporation.

Error assessment

It must be acknowledged that we have found that the results of the plasma exposure of the samples (*e.g.*, the absolute values of concentration of DMPO-OH) were largely affected by small changes in the configuration of the jet, such as the electrodes contact with the quartz tube, the depth of the tube protrusion inside the reactor, and the vertical alignment of the tube. However, while the numerical values changed, the observed trends remained persistent. For example, the concentration of DMPO-OH increased with the initial introduction of H_2O to He feed gas and decreased with higher H_2O content, the concentration of DMPO-OH was lower at 4 mm distance than 10 mm, etc.

Thus, the error assessment was performed within a set configuration of the jet for several conditions (Table S2). We found that conditions of less uniform plasma nature (*i.e.*, in the presence of large amounts of admixtures in the feed gas) generally lead to an increase in standard deviation of the concentration values. The maximum deviation from the mean was found to be *ca.* 12%.

Plasma exposure experiments

In a typical experiment, 100 μL of liquid sample was placed in a well on top of a glass stand inside the reactor. The distance from the nozzle to the sample was 10 mm unless stated otherwise. In experiments when the samples were at the 4 mm distance from the sample to the nozzle, the distance between the live electrode and the sample was maintained at 20 mm (Fig. S3). Thus, the plasma length from the core plasma remained the same throughout all experiments, and the ratio of its quartz surroundings changed (we acknowledge that the plasma jet in contact with quartz will propagate slightly differently than in contact with surrounding gas; nevertheless, this still provided insight into the interaction dynamics with the surrounding atmosphere). The distance between the electrodes was 20 mm in all experiments. The reactor was flushed with the feed gas for 20 s and then exposed to plasma for 60 s.

In spin trapping experiments, a 100 mM solution of a spin trap (PBN, DMPO or DEPMPO) was prepared in H_2O, $H_2{}^{17}O$ or D_2O. Ozone was measured in 60 mM aqueous solutions of TEMP (sodium azide was added in concentrations of 100 mM where stated). In control experiments, solutions of each spin trap were treated with the plasma for the periods of 15, 30, 45 and 60 s. The concentration of each formed radical adduct increased with the plasma treatment time (data not shown).

The experiments involving different feed gas humidity were performed by using split helium flow (*i.e.*, by mixing dry helium with water-saturated helium in desired proportions). Water-saturated helium was made by bubbling dry helium through a water-filled Drechsel flask at 20 °C. The relative humidity was determined by weighing the flask before and after the experiment and comparing the data with the available literature values (Table S3) [*CRC Handbook of Chemistry and Physics*, D. R. Lide (Ed.), 1992, CRC Press, Boca Raton, Florida, USA].

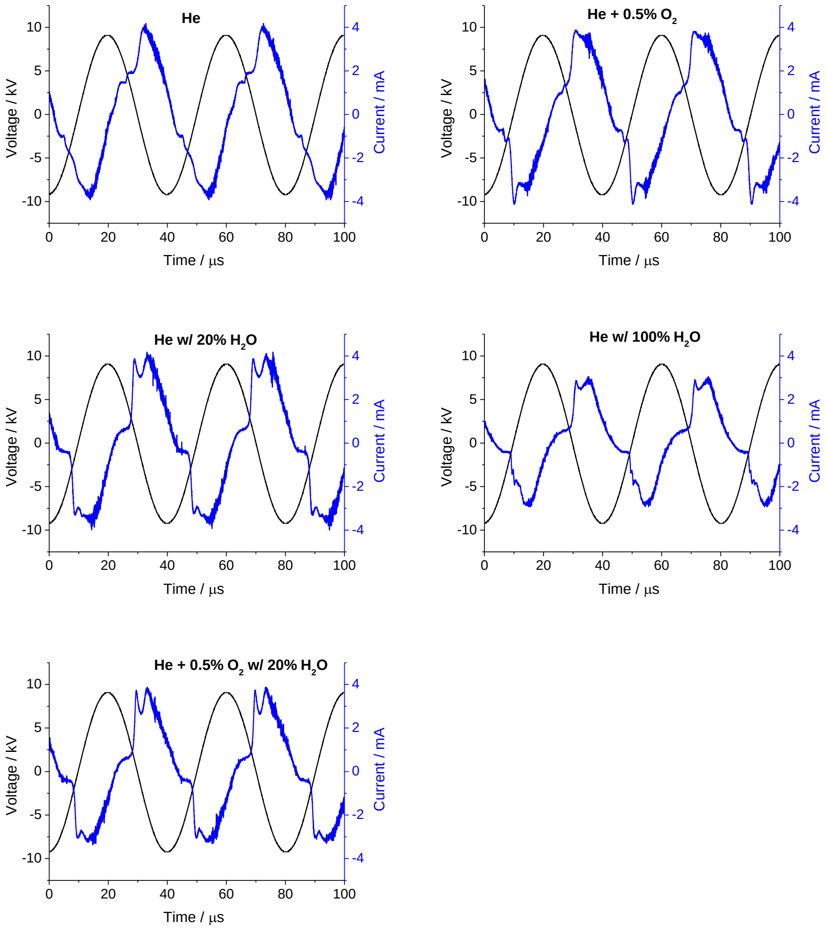

Figure S1. Voltage (black) and return current (blue) waveforms with different feed gas compositions.

Table S1. The results of the OES analysis of the gas phase plasma inside the quartz tube between the electrodes.

Entry	Plasma feed gas	Relative intensity to He signal (706.6 nm)		
		H I (656.2 nm)	O I (777.5 nm)	O I (844.7 nm)
1	He	0.7	5.3	0.5
2	He + 0.5% O_2	0.9	93.1	27.2
3	He + 20% H_2O	12.2	1.2	0.5
4	He + 100% H_2O	9.9	0.5	0.3
5	He + 0.5% O_2 + 20% H_2O	9	6.1	2.3

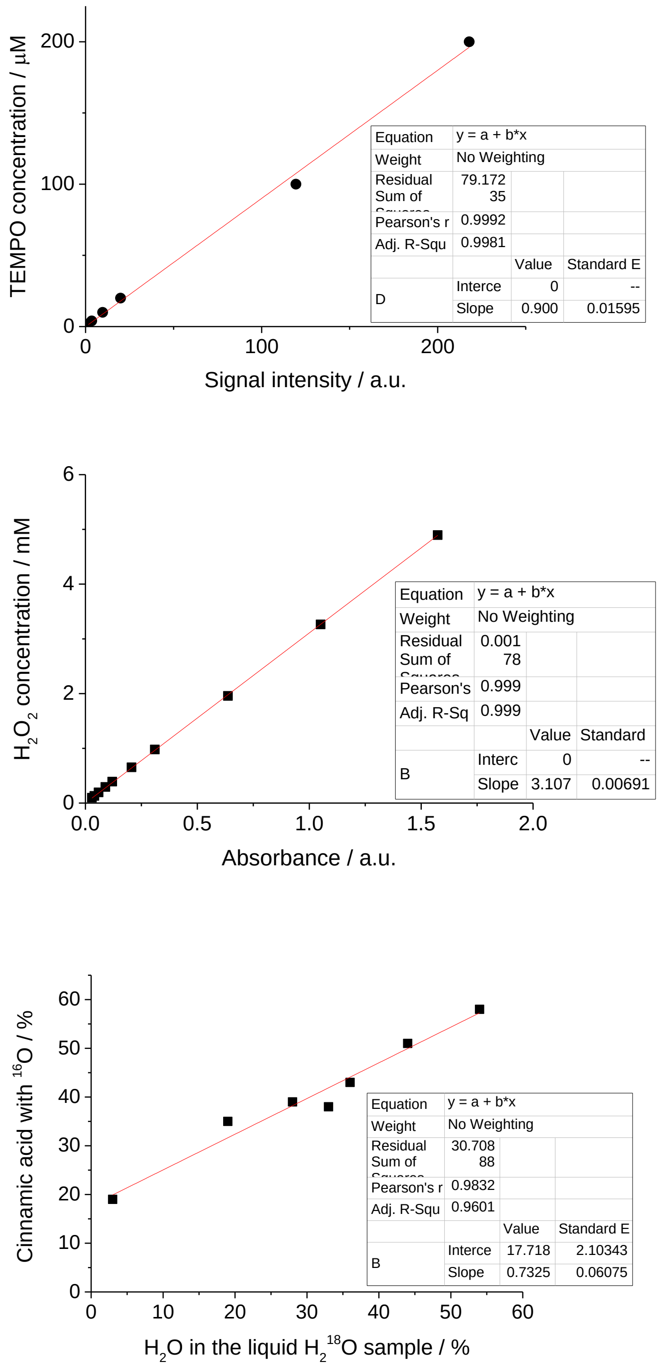

Figure S2. Calibration curve for the analyses of radical adducts using EPR (*top*), H_2O_2 using UV-Vis spectrophotometry (*centre*) and $H_2O/H_2{}^{18}O$ content by MS (*bottom*) in the liquid sample.

Table S2. Error assessment using standard deviation of the concentrations of species in the liquid sample under different conditions.

Entry	Plasma exposure conditions	Species	Concentration in a single experiment (μM)					St. Dev.	
			1	2	3	4	5	μM	%
1	PBN / dry He @ 10 mm	PBN-H	11.8	13.1	11.5	15.2	12.9	1.4	11.2
2	PBN / dry He @ 4 mm	PBN-H	14.0	11.7	14.7	15.3	12.1	1.6	11.6
3	DMPO / He + 0.5%O_2 @ 10 mm	DMPO-OH	13.8	12.5	15.2	15.0	13.5	1.1	7.8
4	DMPO / He + 50%H_2O @ 10 mm	DMPO-OH	9.6	11.3	10.4	11.4	11.8	0.9	8.2
5	TEMP / He + 0.5%O_2 + 60%H_2O @ 10 mm	TEMPO	14.6	12.7	13.6	15.3	16.5	1.5	10.1
6*	He + 50%H_2O @ 10 mm	H_2O_2	1.6	1.5	1.5	1.4	1.6	0.1	5.7

*Concentration and st.dev. shown in mM.

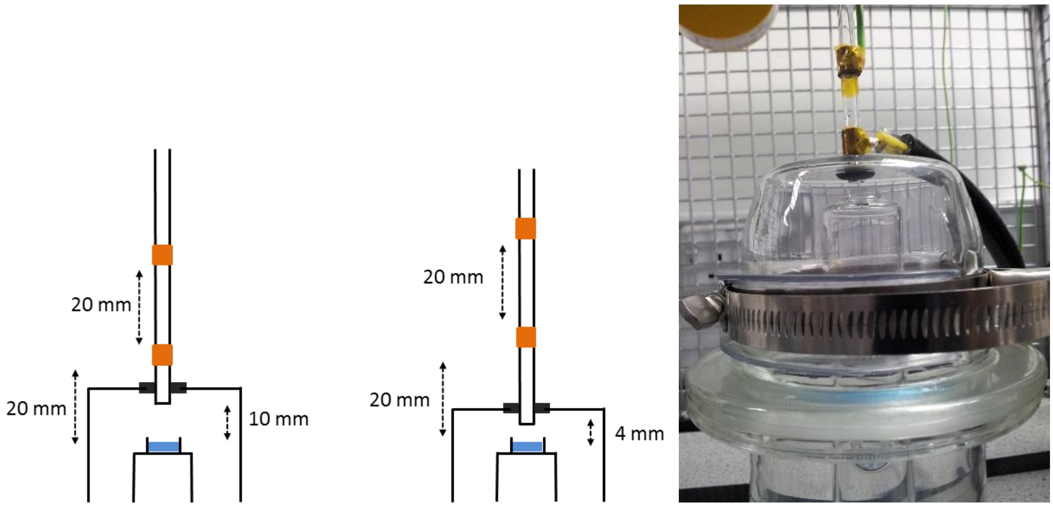

Figure S3. A schematic representation of the experimental conditions of 4 and 10 mm from the nozzle to the liquid sample and a photo of the experimental setup.

Table S3. H_2O concentration in the feed gas with helium passing through a Drechsel flask filled with H_2O.

Time (min)	Final H_2O temperature (°C)	H_2O content of the gas (μg/L)	
		Experimental*	Calculated**
5	20	17.4	17.3
17	19.6	17.0	17.1
20	19.5	16.9	17.1
60	18.4	16.8	16.5

*Based on the weight of evaporated H_2O.
**Averaged for the temperature interval; based on the data available in literature for 100% H_2O vapour saturation of gases.

Table S4. H_2O content in the liquid $H_2{}^{17}O$ sample after plasma exposure.

Entry	H_2O vapour saturation of the feed gas (%)	Distance* (mm)	$H_2{}^{16}O$ content of the liquid sample*** (%)
1**	-	-	18
2	-	10	18.5
3	10	10	24.5
4	10	4	22

*Distance from the nozzle to the sample.
**Amount of $H_2{}^{16}O$ found in pristine $H_2{}^{17}O$.
***Averaged value for 2-3 measurements.

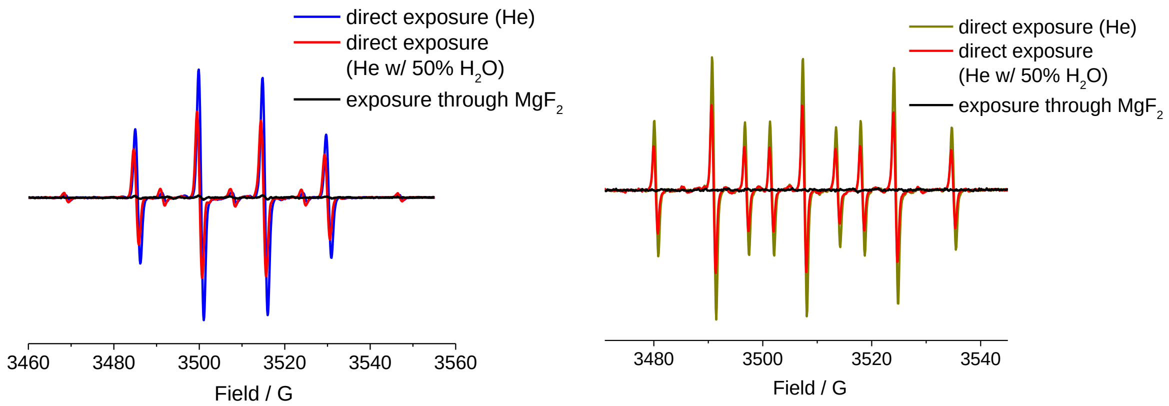

Figure S4. EPR spectra of DMPO (*left*) and PBN (*right*) spin adducts obtained in experiments with direct plasma exposure of the sample and through the magnesium fluoride window.

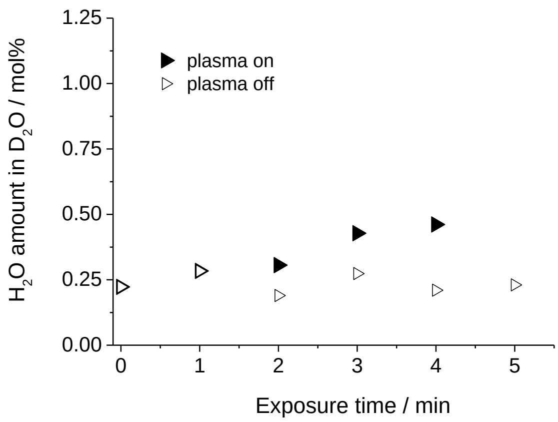

Figure S5. H_2O amount in a D_2O liquid sample delivered by helium flow with no added water vapour (▷) and helium flow with ignited plasma (▶) as determined by ^{1}H NMR.

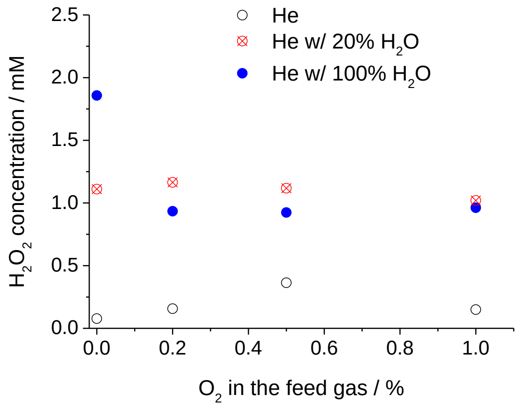

Figure S6. H_2O_2 concentration in the liquid as a function of O_2 content of the feed gas with dry He (○), He with 20% (⊗) and He with *ca.* 100%* (●) H_2O vapour saturation.

*In this case the H_2O vapour saturation was 100, 99.8, 99.5 and 99% for 0, 0.2, 0.5 and 1% O_2 admixtures, respectively. However since the difference did not exceed 1%, it is referred to as 100% henceforth.

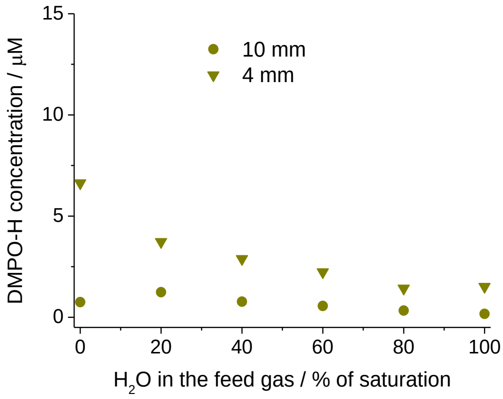

Figure S7. DMPO-H adduct concentration in the liquid sample at 10 mm (●) and 4 mm (▼) distance from nozzle to sample as a function of H_2O vapour saturation of the feed gas with no added oxygen.

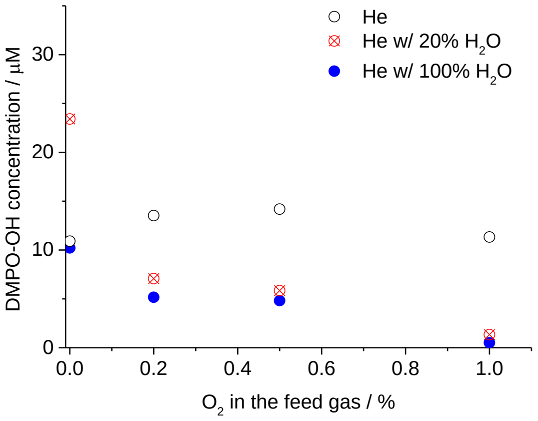

Figure S8. DMPO-OH adduct concentration in the liquid as a function of O_2 content of the feed gas with dry He (○), He with 20% (⊗) and He with *ca.* 100% (●) H_2O vapour saturation.

Table S5. Experimental investigation of primary kinetic isotope effect in the case of H_2O/D_2O as sources of H/D radicals.

Entry	Introduced isotopes relative amount* (mol%)		Adduct relative amount** (%)		KIE***
	H_2O	D_2O	H	D	
1	5	95	14.1	85.9	3.1
2	10	90	23.0	77.0	2.7
3	20	80	45.2	54.8	3.3
				average value	3.0

*Percentage shown corresponds to both initial liquid and vapour composition.
**The values shown are calculated as a percentage of total hydrogen isotopes adducts of PBN (PBN-H and PBN-D).
*** Calculated as shown in Eq. S1.

Primary kinetic isotope effect factor was calculated using the following equation:

$$\mathbf{KIE} = \frac{\mathbf{H}(\%) \cdot \mathbf{D_2O}(\mathbf{mol}\%)}{\mathbf{H_2O}(\mathbf{mol}\%) \cdot \mathbf{D}(\%)} \qquad (\mathbf{S1})$$

where KIE is the primary kinetic isotope effect factor, H(%) and D(%) are the relative amounts of formed PBN-H and PBN-D adducts, H_2O(%) and D_2O(%) are the relative molar amounts of introduced H_2O and D_2O (in both feed gas vapour and liquid sample), respectively.

Here, the KIE calculations were performed under assumption that H and D radicals diffuse into the liquid from the gas phase with the same rate. Realistically, in a hypothetical situation when same amounts of H and D are formed in the gas phase, (2/1)^0.5 = 1.4 times less D atoms will reach the liquid sample surface in the same period of time. However, for radical species some other factors change as well, such as recombination rate. Importantly, the calculated KIE is the 'apparent KIE', *i.e.* the actual experimental KIE without deconvolution into vibrational energy KIE and other factors.
Noteworthy, in the case of D_2O/H_2O the said law implies only a minor difference ((18/16)^0.5). While this must be acknowledged, the experimental data (see the article text, Table 1) showed that this effect could be disregarded.

Table S6. Concentrations and relative amounts of PBN-H and PBN-D radical adducts after plasma exposure with H_2O and D_2O in the feed gas and the liquid sample.

Entry	Experimental conditions		Adduct concentration** (μM)		Adduct relative amount*** (%)	
	Distance* (mm)	Feed gas vapour saturation (%)	**H**	**D**	**H**	**D**
		**D_2O liquid / D_2O vapour**				
1		-	1.9	8.3	19	81
2	10	10	0.4	9.8	4	96
3		100	0.0	4.1	-	>99
4		-	4.3	6.6	40	60
5	4	10	0.3	10.4	2	98
6		100	-	4.6	-	>99
		**H_2O liquid / H_2O vapour**				
7		-	12.9	-	>99	-
8	10	10	11.9	-	>99	-
9		100	4.2	-	>99	-
10		-	13.6	-	>99	-
11	4	10	11.1	-	>99	-
12		100	4.3	-	>99	-

*Distance from the nozzle to the sample.
**Additional PBN adducts such as *e.g.* PBN-OH were also detected (data not shown).
***The values shown are calculated as a percentage of the total amount of hydrogen isotopes adducts of PBN (PBN-H and PBN-D).

Table S7. Experimental and calculated relative amount of PBN adducts.

Entry	Liquid composition* (mol%)		PBN adduct relative amount**(%)			
			Calculated***		Experimental****	
	H_2O	D_2O	H	D	H	D
1	0.3	99.7	1	99	17	83
2	1.9	98.1	5	95	78	22
3	13.5	86.5	32	68	93	7
4	86.2	13.8	95	5	18	82

* Based on the ^{1}H NMR data (see Table 1).
** The values shown are calculated as a percentage of total hydrogen isotopes adducts of PBN (PBN-H and PBN-D).
*** Calculated under assumption that radicals originate in the liquid phase; Eq. S2, S3.
****Calculated based on the data from Table 2.

$$\mathrm{H}(\%) = \frac{\mathrm{KIE} \cdot \mathrm{H_2O}(\%) \cdot 100}{\mathrm{D_2O(mol\%)} + \mathrm{KIE} \cdot \mathrm{H_2O(mol\%)}} \qquad \text{(S2)}$$

$$\mathrm{D}(\%) = 100 - \mathrm{H}(\%) \qquad \text{(S3)}$$

where H(%) and D(%)are the relative amounts of formed PBN-H and PBN-D adducts, KIE is the primary kinetic isotope effect factor, H_2O(%) and D_2O(%) are the relative molar amounts of the H_2O and D_2O **in the liquid sample**, respectively (see the main article text, Table 1).
The calculations here are based on the approximation that the composition of the liquid sample (D_2O + delivered H_2O) remained the same throughout the plasma exposure experiment. The real value of H_2O mol% in the D_2O sample at any moment of exposure was lower.

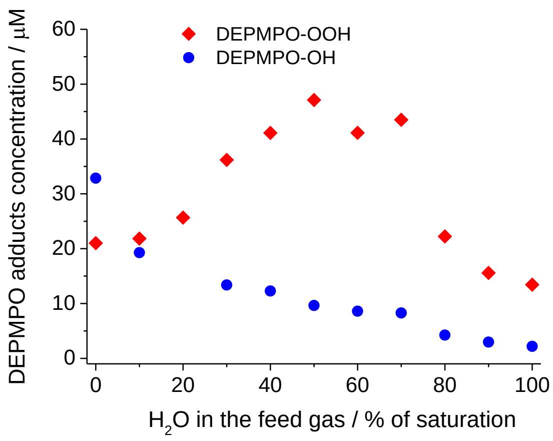

Figure S9. DEPMPO-OOH (◆) and DEPMPO-OH (●) radical adduct concentration in the liquid as a function of H_2O vapour saturation of the feed gas with 0.5% O_2.

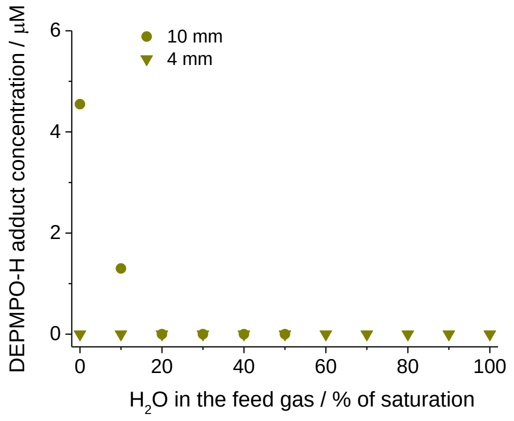

Figure S10. The concentration of the DEPMPO-H adduct as a function of H_2O vapour saturation of the feed gas with no added O_2 at 10 mm (●) and 4 mm (▼) distance from the nozzle to the sample.

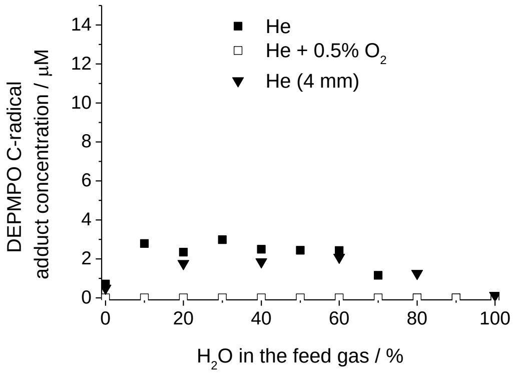

Figure S11. The concentration of the DEPMPO adduct of a carbon-centred radical in the liquid as a function of H_2O vapour saturation of the feed gas with He (■) and He + 0.5% O_2 (□) at 10 mm and He (▼) at 4 mm distance from the nozzle to the sample.

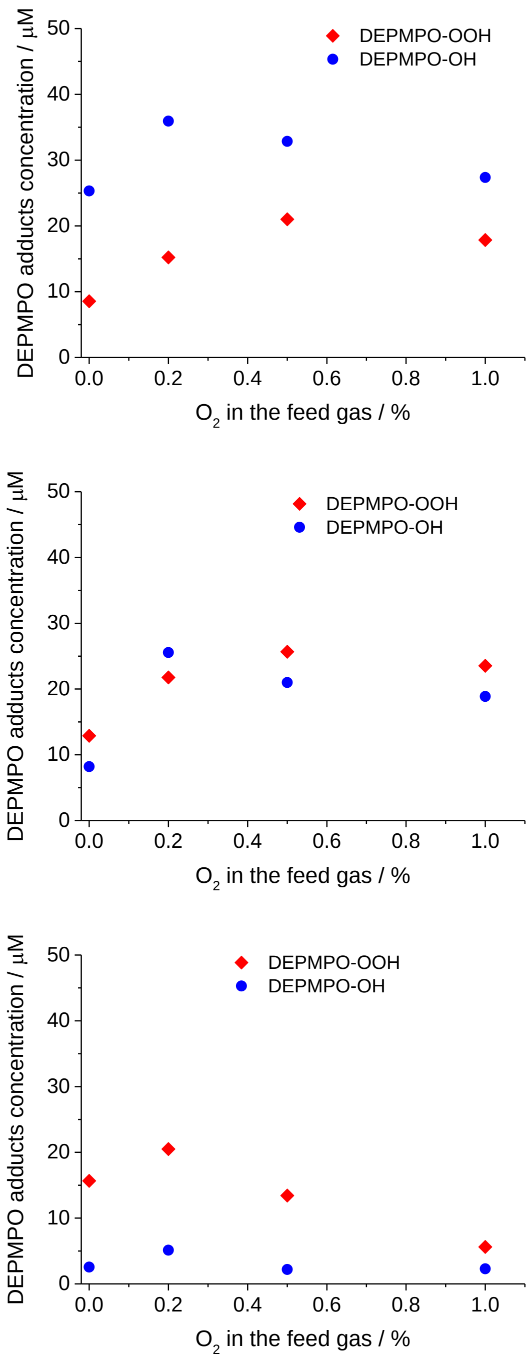

Figure S12. DEPMPO-OOH (◆) and DEPMPO-OH (●) adducts concentration in the liquid as a function of the O_2 content of the feed gas with dry He (*top*), He with 20% (*centre*) and He with 60% (*bottom*) H_2O vapour saturation.

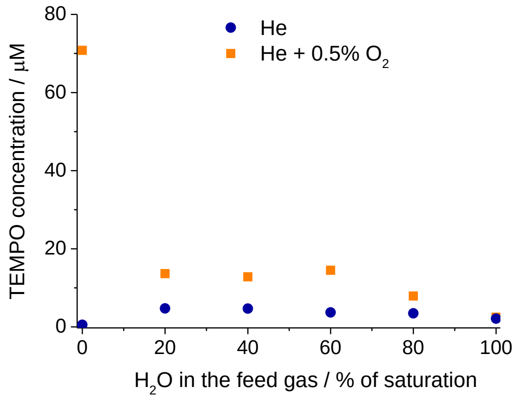

Figure S13. Concentration of the formed TEMPO in aqueous solutions of TEMP with helium (●) and helium with 0.5% oxygen admixture (■) as a function of the H_2O vapour saturation of the feed gas.

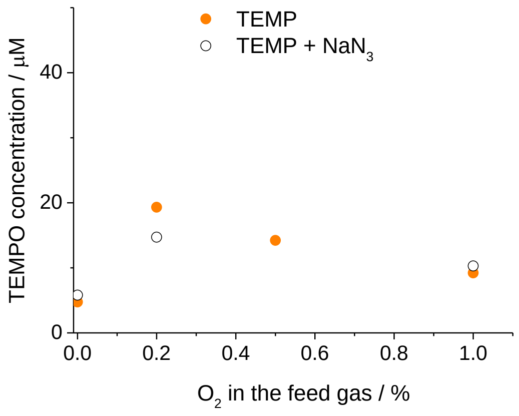

Figure S14. Concentration of the formed TEMPO in aqueous solutions of TEMP (●) and TEMP with added sodium azide (○) as a function of the O_2 content of the feed gas with 20% H_2O vapour saturation.
